# G-protein signaling is required for increasing germline stem cell division frequency in response to mating in *Drosophila* males

**DOI:** 10.1038/s41598-020-60807-8

**Published:** 2020-03-03

**Authors:** Manashree S. Malpe, Leon F. McSwain, Karl Kudyba, Chun L. Ng, Jennie Nicholson, Maximilian Brady, Yue Qian, Vinay Choksi, Alicia G. Hudson, Benjamin B. Parrott, Cordula Schulz

**Affiliations:** 1 Department of Cellular Biology, University of Georgia, Athens, GA 30602 USA; 2 Winship Cancer Institute, Emory University, Atlanta, GA 30322 USA; 3 University of Texas Southwestern Medical Center, Dallas, TX USA; 4 University of North Georgia, Department of Biology, Oakwood, GA 30566 USA; 5 School of Medicine, Duke University, Durham, NC 27708 USA; 6 Odum School of Ecology, University of Georgia, Athens, GA 30602 USA

**Keywords:** Cell proliferation, Stem cells

## Abstract

Adult stem cells divide to renew the stem cell pool and replenish specialized cells that are lost due to death or usage. However, little is known about the mechanisms regulating how stem cells adjust to a demand for specialized cells. A failure of the stem cells to respond to this demand can have serious consequences, such as tissue loss, or prolonged recovery post injury. Here, we challenge the male germline stem cells (GSCs) of *Drosophila melanogaster* for the production of specialized cells, sperm cells, using mating experiments. We show that repeated mating reduced the sperm pool and increased the percentage of GSCs in M- and S-phase of the cell cycle. The increase in dividing GSCs depended on the activity of the highly conserved G-proteins. Germline expression of RNA-Interference (RNA-*i*) constructs against G-proteins, or a dominant negative G-protein eliminated the increase in GSC division frequency in mated males. Consistent with a role for the G-proteins in regulating GSC division frequency, RNA-*i* against seven out of 35 G-protein coupled receptors (GPCRs) within the germline cells also eliminated the capability of males to increase the numbers of dividing GSCs in response to mating.

## Introduction

Metazoan tissues undergo homeostasis wherein stem cells divide and their daughter cells proliferate and differentiate to replace lost cells. The human hematopoietic stem cells, for example, renew a remarkable number of about one trillion blood cells per day^1,2^. Stem cells have to maintain a baseline mitotic activity for the production of daughter cells that account for the daily turnover of differentiated cells. However, whether stem cells can modulate their mitotic activity in response to demands that challenge the system is not fully explored. In some instances, stem cells respond to physiological cues; for example, murine hematopoietic stem cells divide more frequently during pregnancy due to increased oestrogen levels^3^. In *Drosophila melanogaster*, intestinal stem cells initiate extra cell divisions upon ablation of differentiated gut cells, and GSCs modulate their mitotic activity in response to environmental conditions, such as nutrient availability and temperature^4–7^.

*Drosophila* is an excellent model for identifying the molecules and mechanisms that regulate and fine-tune tissue homeostasis. A plethora of genetic tools are available for manipulating and monitoring dividing adult stem cells. The small size of the fly, the short generation cycle, and the fairly low costs covering their maintenance allow for high throughput screens. Here, we subjected several thousand male and several million virgin female flies to mating experiments, a task challenging to perform with vertebrate model organisms. We discovered that repeated mating caused a reproducible and significant increase in GSC division frequency in *Drosophila wild-type* (*wt*) males. Our analysis revealed that this response to mating was dependent on the activity of G-proteins. Impairing G-protein activity from the germline cells eliminated the ability of the GSCs to increase their division frequency in response to mating.

G-proteins are highly conserved molecules that associate with GPCRs. GPCRs constitute a large family of cell surface receptors that mediate the cell’s response to a wide range of external stimuli, including odors, pheromones, hormones, and neurotransmitters. Loss of GPCR signaling affects countless developmental and neural processes in humans, as well as vertebrate and invertebrate model organisms^8–10^. Here we show that reducing the expression of seven out of 35 GPCRs via RNA-*i* from the germline cells eliminated the capability of males to increase their GSC division frequency when mated. These were the Serotonin (5-HT) Receptors 1 A, 1B and 7, Methuselah (Mth), Methuselah-like5 (Mth-l5), Octopamineβ2-Receptor (Octβ2R), and a predicted GPCR encoded by *CG12290*.

A role for any of these GPCRs in regulating GSC division frequency is novel. No previous study has identified any functional role for Mth-l5 or CG12290. Serotonin, Octopamine, and Mth signaling play opposing roles in life-span, locomotion, and sleep^11–16^. Mth signaling also regulates vesicle trafficking at the synapse, Octopamine signaling regulates ovulation, and Serotonin signaling plays essential roles in memory formation and learning^17–19^.

## Results

### Mating increased the percentage of GSCs in M-phase of the cell cycle

**Figure 1:**
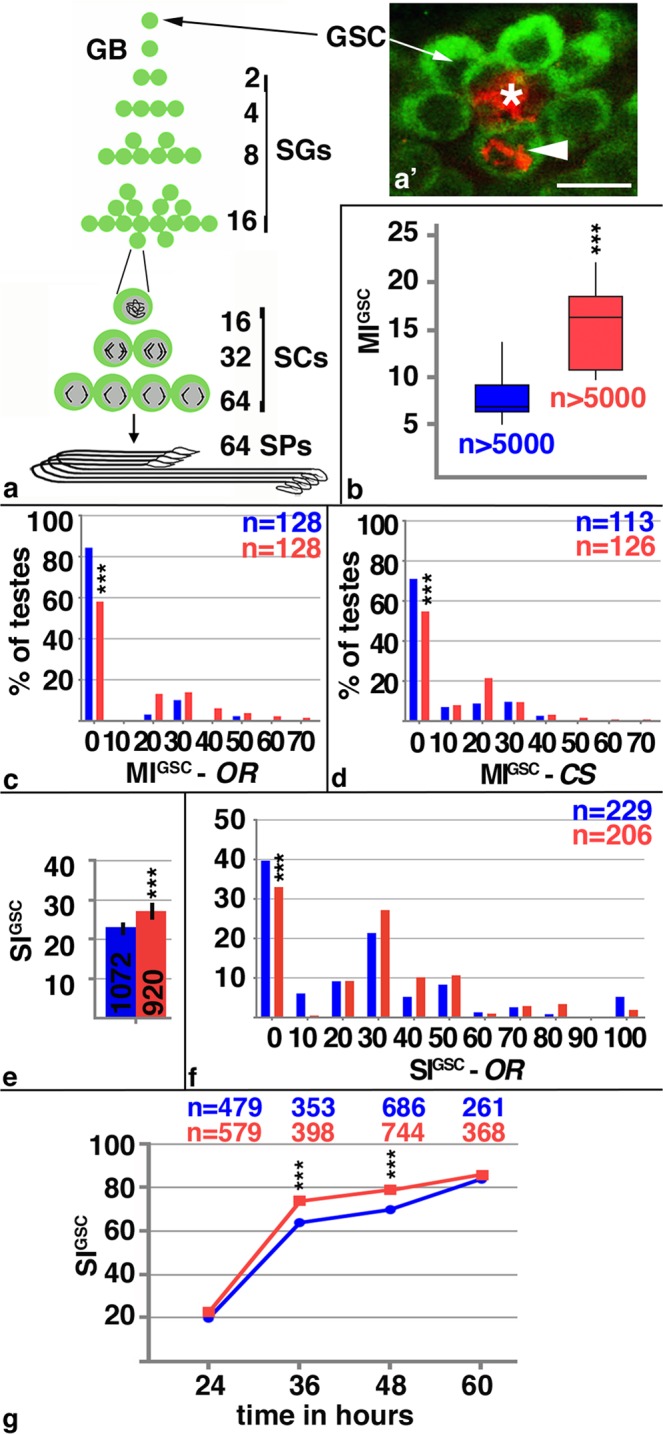
Mating increased male GSC division frequency. (**a**) Cartoon depicting the stages of *Drosophila* spermatogenesis. Note that every GSC division produces exactly 64 spermatids. GB: gonialblast, SG: spermatogonia, SC: spermatocytes, SP: spermatids. (a’) The apical tip of a *wt* testis. The FasIII-positive hub (asterix) is surrounded by seven Vasa-positive GSCs (green), one of which is in mitosis based on anti-pHH3-staining (arrowhead). Scale bar: 10 μm. (**b–g**) Blue: non-mated condition, red: mated condition, ***P-value < 0.001, numbers of GSCs and number of gonads (n=) as indicated. (**b**) Box plots showing the range of MI^GSC^. Lines within boxes represent medians, whiskers represent outliers. (**c,d**) FDGs showing bin of MI^GSC^ (bin width = 10) across a population of (**c**) *OR* and (**d**) *CS* males on the X-axis and the percentage of testes with each MI^GSC^ on the Y-axis. (**e**) Bar graph showing SI^GSC^ of *OR* males from three independent experiments. (**f**) FDGs showing bin of SI^GSC^ (bin width = 10) across the population of *OR* males on the X-axis and the percentage of testes with each SI^GSC^ on the Y-axis. (**g**) Graph showing the percentage of EdU-marked *OR* GSCs on the Y-axis and hours of feeding and mating on the X-axis.

As is typical for many stem cells, the *Drosophila* GSCs are found in a specific cellular microenvironment. They are located at the tip of the gonad, where they are attached to somatic hub cells (Fig. 1a,a’). Upon GSC division, one of the daughter cells, called the gonialblast, undergoes four rounds of stem cell daughter characteristic, transit-amplifying divisions, resulting in 16 spermatogonia. Subsequently, spermatogonia enter a tissue-specific differentiation process. They grow in size, undergo the two rounds of meiosis, and develop through extensive morphological changes into elongated spermatids^20^. According to this tightly controlled homeostasis program, each GSC division can only produce 64 spermatids (Fig. 1a). Thus, an increase in sperm production is reliant on the GSCs.

We investigate division frequency using an established immunofluorescence protocol^7^. In this approach, Vasa-positive GSCs are identified based on their position adjacent to FasciclinIII (FasIII)-positive hub cells (Fig. 1a’). The percentage of GSCs in mitosis, the M-phase index (MI), is investigated by staining against the mitosis marker, phosphorylated Histone-H3 (pHH3). The MI of the GSCs (MI^GSC^) is calculated by dividing the number of pHH3-positive GSCs by the total number of GSCs.

To investigate if stem cells can modulate their division frequency in response to a demand for specialized cells, we challenged *Drosophila* males in mating experiments. For each experiment, 80–100 males were exposed individually to virgin females. An equal number of male siblings were each kept in solitude and served as the non-mated controls. To keep experimental variation to a minimum, we employed a three-day mating protocol for all experiments, kept the animals under the same conditions, dissected the testes at the same time of the same day, and dissected experimental groups in tandem. Using *wt* males, we obtained robust and reproducible increases in MI^GSC^ in response to mating. The box-plot in Fig. 1b shows the observed difference in MI^GSC^ between mated and non-mated populations of isogenized *wt*, *Oregon R* (*OR*), males from 17 independent mating experiments. Interestingly, we observed variability in MI^GSC^ among males of each condition. The MI^GSC^ of non-mated males ranged from six to nine percent, with a median at seven percent. The MI^GSC^ of mated males ranged from 11 to 18 percent, with a median at 16.5 percent. We hypothesize that this variability in MI^GSC^ within each condition is due to naturally occurring physiological differences within the flies. Although the increase in MI^GSC^ in response to mating varied among the different experiments, it was always biologically and statistically significant.

While imaging and analyzing the tissue, we noted that several testes from mated males contained multiple GSCs positive for pHH3. Therefore, we wondered whether the difference in MI^GSC^ between non-mated and mated males seen in Fig. 1b is caused by the presence of only a few testes with an extremely high MI^GSC^. As the box blot does not provide information how the dividing GSCs are distributed across all testes, we analyzed our data in a less processed format, using frequency distribution graphs (FDGs). FDGs show how often a particular value is represented within a population. In our case, they group the data by MI^GSC^ (or synthesis phase index, SI^GSC^) for each testis. For all FDGs shown, the x-axis separates the testes in bins of ten; for example, a bin with the median of 20 plotted on the x-axis contains all testes with indices ranging from 15 to 25. The y-axis shows the percentage of testes for each bin.

The FDG of one population of *OR* flies revealed that most testes had an MI^GSC^ of zero, while the others had MIs^GSC^ that ranged up to 70 (Fig. 1c). Notably, calculating the MI^GSC^ per testis yields much higher values than averaging the MI^GSC^ for the whole population of testes, as the latter includes the many MIs^GSC^ of zero (compare FDG in Fig. 1c to box blot in Fig. 1b). The FDGs show that mated males had significantly fewer testes with an MI^GSC^ of zero and many testes with a higher MI^GSC^ compared to non-mated siblings (Fig. 1c). We observed the same result for another isogenized *wt* strain, *Canton S* (*CS*, Fig. 1d). We conclude that mating affected the MI^GSC^ of many males within one mated population.

### Exposure to virgin females, courtship, and single mating events do not appear sufficient to increase MI^GSC^

Next, we wondered if exposure to females, or courtship behavior is sufficient to increase MI^GSC^. The *Drosophila* mating behavior follows a genetically controlled series of events, during which the animals have visual, chemical, and physical contact^21^. Specifically, cuticular hormones on the body of the flies serve as chemical cues for the identification of gender and mating status^22^. To investigate if these are sufficient to increase MI^GSC^, we exposed *OR* males to either *OR* male or *OR* virgin female cuticular hormone extracts. We investigated MI^GSC^ after three days of daily treatment. While our positive control, mated *OR* males (Supplemental Fig. S1a, M + F), displayed a significantly higher MI^GSC^ compared to non-mated *OR* males (Supplemental Fig. S1a, M + M), no significant increase in MI^GSC^ was observed in *OR* males that were exposed to male cuticular hormone extract (Supplemental Fig. S1a, M + MCH), or in *OR* males that were exposed to *OR* virgin female cuticular hormone extract (Supplemental Fig. S1a, M + FCH) compared to each other and to the non-mated control. Next, we allowed the flies to see, smell, and touch each other. For this, we used a feeding construction (Supplemental Fig. S1a’) that separates *OR* males from other *OR* males or from *OR* virgin females. Again, no significant increase in MI^GSC^ was observed in neither males that were net-separated from other males (Supplemental Fig. S1a, M net M) nor in males net-separated from virgin females (Supplemental Fig. S1a, M net F) for three days compared to each other and to the non-mated control. Finally, we housed males for three days with decapitated *OR* virgin females, a situation that allows the males to see and smell the virgin females, and interact more closely with the female virgin body than in the net-separated situation. Still, no significant increase in MI^GSC^ was observed (Supplemental Fig. S1a, M + decap F). We conclude that vision, smell, and touch are not sufficient to increase MI^GSC^.

Males that are mutant for the *fruitless* (*fru*) gene cannot distinguish between males and females, and thus court either gender. The *fru*^1^ and *fru*^3^ alleles, as well as *fru*^3^*/fru*^4–40^ were previously shown to display male-to-male courtship behavior and another *fru* allele, *fru*^*P1LexA*^, acts as a genetic null mutation^23–25^. When males from these allelic combinations were housed together, we observed them chaining each other and flapping their wings, indicative of courtship. However, their MIs^GSC^ were not increased (Supplemental Fig. S1b, purple bars) compared to single housed *fru* mutant males (Supplemental Fig. S1b, blue bars). This observation suggests that courtship alone is also not sufficient to increase MI^GSC^. In summary, we did not observe an increase in MI^GSC^ in any of the above situations, strongly suggesting that the males actually have to mate with the females in order for an increase in MI^GSC^ to occur.

Finally, we housed *OR* males to varying numbers of *OR* virgin females and subsequently analyzed how many of their GSCs were in mitotic division. When we exposed *OR* males for 24 hours to one (1 F, 24 hrs), two (2 F, 24 hrs), or three (3 F, 24 hrs) female virgins, no significant difference in MI^GSC^ between non-mated and mated males was apparent (Supplemental Fig. S1c). After 24 hours of mating, the females were placed into one food vial each and investigated a few days later for the appearance of progeny. 95% of the single and pairs of females, and 72% of the triplets of females exposed to males produced offspring, indicating that the lack of increase in MI^GSC^ was not due to a failure of the males to mate within the first 24 hours.

Robust and reproducible increases in MIs^GSC^ were seen in *OR* males that were exposed to three virgin females on each of two (2 × 3 F, 48 hrs) or three (3 × 3 F, 72 hrs) days of mating (Supplemental Fig. S1c). Although the true number of times each male mated is inconclusive, our data strongly suggest that males have to mate repeatedly with virgin females for an increase in MI^GSC^ to become apparent. The increase in MI^GSC^ in mated males was reversible, showing that the response to mating was dynamic. Moving males back into solitude after the three-day mating experiment (3 × 3 F, 120 hrs) eliminated the increase in MI^GSC^ (Supplemental Fig. S1c). Control males that continued to mate (5 × 3 F, 120 hrs), in contrast, still had a significant increase in MI^GSC^ (Fig. S1c).

### Mating increased the percentage of GSCs in S-phase of the cell cycle

As another measure of cell divisions, we investigated the percentage of GSCs in synthesis phase (S-phase) of the cell cycle. Testes were labeled with 5-ethynyl-2′-deoxyuridine (EdU) and the SI^GSC^ was calculated by dividing the number of EdU-positive GSCs by the total number of GSCs. Using pulse-labeling experiments, we observed that mated *OR* males displayed significant higher SI^GSC^ compared to their non-mated siblings (Fig. 1e). A FDG shows that the changes in SI^GSC^ affected many males within the population (Fig. 1f).

In an attempt to investigate how mating affects the G-phases of the cell cycle, we employed the Fly-FUCCI technology in combination with the UAS-Gal4 expression system^26–28^. With Fly-FUCCI, the coding regions of fluorescent proteins are fused to the destruction boxes of cell cycle regulators, allowing the marking of different cell cycle stages. These artificial genes are expressed under control of the Yeast Upstream Activating Sequences (UAS)^26^. Their expression can be induced using tissue-specific Gal4-transactivators^27,28^. For our experiments, we used a *nanos-Gal4*-transactivator (NG4) with a reported expression in GSCs, gonialblasts, and spermatogonia^29^. Unfortunately, this approach was not useful for our study. We expressed the FUCCI-constructs from two independent lines within the male germline cells, but were not able to observe a response to mating, based on anti-pHH3 staining. While mated control animals (*Fucci/wt*) increased their MIs^GSC^ compared to their non-mated siblings, no increase in MIs^GSC^ was observed in mated compared to non-mated *Fucci/NG4* males (Supplemental Fig. S2a). One possible explanation for this could be that the expression of proteins with destruction boxes overloaded the cell cycle machinery of male GSCs.

Our data suggest that GSCs in mated males either undergo a shorter cell cycle or that within these GSCs, M- and S-phases occupy a larger proportion of the cell cycle compared to the GSCs in non-mated controls. Previous research has used incorporation of a thymidine analog to investigate GSC cell cycle length. It was reasoned that if the GSCs within one genotype, or under one condition cycle faster, then it would take less time for the GSCs in the experimental animals to be positively labeled for the thymidine homologue compared to the GSCs in control animals^7,30^. Following this reasoning, we fed *OR* animals EdU during the mating experiment. We then calculated how many GSCs had been in S-phase at different time points. Our EdU-incorporation experiment revealed that the number of EdU-positive GSCs increased rapidly after 24 hours of feeding, and reached 80% at 60 hours of feeding (Fig. 1g). Prolonged feeding further increased the numbers of EdU-positive GSCs, but this data was excluded from the study as the majority of males that were fed EdU while mating had died by 72 hours of the experiment. Most importantly, mated males had significantly more EdU-positive GSCs at 36 and 48 hours of mating compared to their non-mated siblings (Fig. 1g). This experiment confirms the increase in SI^GSC^ from our pulse chase experiment and shows that, in mated males, more GSCs had entered S-phase of the cell cycle.

### Mating reduced the sperm pool

To confirm that our mating experiments created a demand for sperm, we explored differences in the sperm pool of the seminal vesicles between non-mated and mated males. For this, we used two different transgenic constructs that label the sperm. A Don Juan-Green Fluorescent Protein (DJ-GFP) reporter labels the sperm bodies and allows to assess the overall amount of sperm within the seminal vesicles^31^. A ProtamineB-GFP (Mst35B-GFP) line only labels the sperm heads and can be used to count individual sperm within the seminal vesicles^32^. With each of these reporters, individualized mature sperm was normally seen within the seminal vesicle of the male reproductive tract.

**Figure 2:**
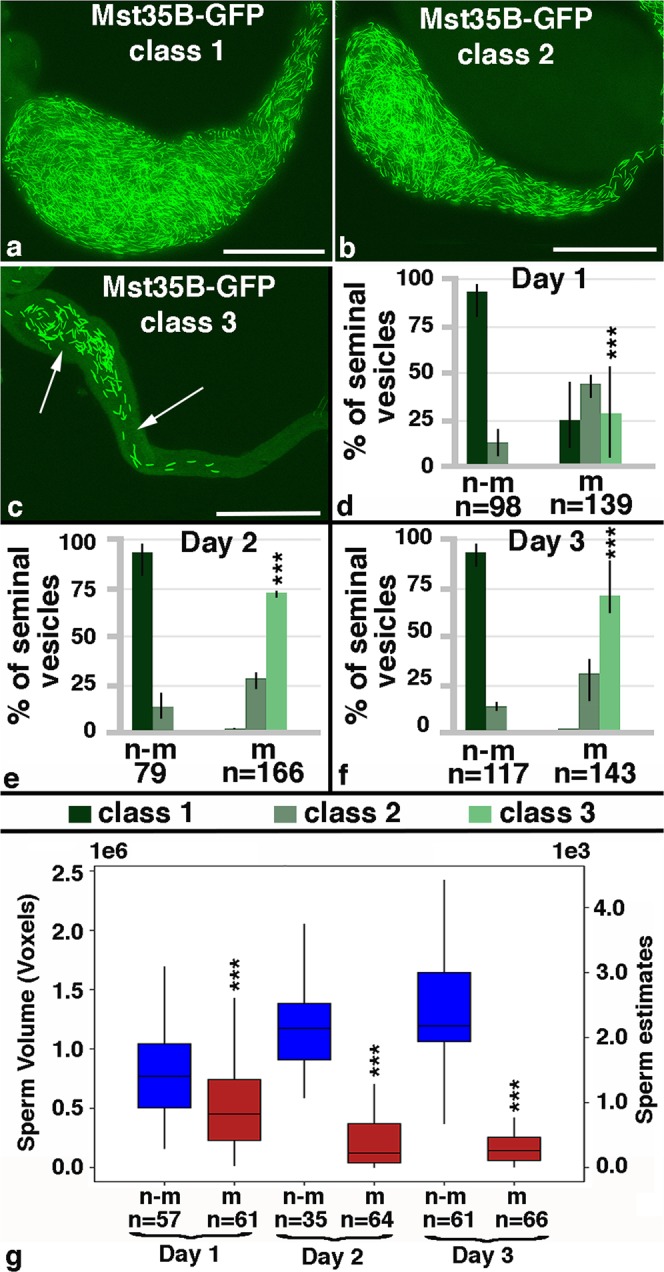
Mating reduced the mature sperm pool. (**a–c**) Class 1, 2 and 3 seminal vesicles from Mst35B-GFP males. Scale bars: 0.1 mm; arrows point to GFP-negative regions. (**d–g**) Numbers of seminal vesicles (n=) as indicated, n-m: non-mated, m: mated, ***P-value < 0.001. (**d–f**) Bar graphs showing the distribution of Class 1 to 3 seminal vesicles in non-mated and mated males at days one to three of the mating experiment. Three fly lines that carry GFP-marked sperm were used: one that carries Dj-GFP (BL#5417), one that carries the Mst35B-GFP (BL#58408), and one line that carries both constructs (BL#58406). g) Box plot showing sperm head volume and estimate of sperm numbers (based on MST35B-GFP) per seminal vesicle in non-mated and mated males on days one to three of the experiment.

According to the literature, the total number of sperm within one seminal vesicle varies among different *Drosophila* species and among genetic backgrounds^32–34^. To keep the genetic background consistent among our experiments, we crossed each of the reporter lines to *OR* females and used their male progeny for our experiments. The seminal vesicles were then analyzed at days one to three of the experiment. Based on the size and fluorescence of the seminal vesicles, we first sorted them into three classes. Class 1 and class 2 seminal vesicles were completely filled with GFP-positive sperm heads; however, class 1 seminal vesicles were very wide (Fig. 2a), while class 2 seminal vesicles were thinner (Fig. 2b). Class 3 seminal vesicles contained only few GFP-positive sperm and had areas that were not filled with GFP (Fig. 2c, arrows). A qualitative examination revealed that non-mated males had mostly class 1 and 2 seminal vesicles, while mated males had mostly class 3 seminal vesicles. We still detected a fair number of class 1 and 2 seminal vesicles in males that had mated for only one day, but these were severely reduced in males after two and three days of mating (Fig. 2d–f). Using the area function of the ImageJ software, we confirmed the changes in the size of the seminal vesicles upon mating. Males that were mated for three days had a significantly lower surface area of their seminal vesicles compared to their non-mated siblings (Supplemental Fig. S2b)

To validate that mating reduces the amount of sperm, we developed an automated procedure. This allowed us to investigate larger numbers of seminal vesicles compared to a previously reported method, in which images through the seminal vesicles were flattened and sperm heads counted by eye^32^. Furthermore, a computer-based calculation eliminates subjective bias introduced by the investigator. The computer program calculates the volume occupied by Mst35B-GFP-positive sperm heads per seminal vesicle in all focal planes (voxels in Fig. 2g). Based on this technique, the sperm heads of mated males occupied significantly less volume within the seminal vesicles than the sperm heads in non-mated males (Fig. 2g). Notably, the total volume occupied by sperm became more reduced with every day of mating. By days two and three of mating it ranged from 0.1 to 0.4 ×10^6^ voxels per seminal vesicle. The non-mated sibling controls, in contrast, maintained a large GFP-occupied volume in their seminal vesicles, with an average of 1.2 × 10^6^ voxels per seminal vesicle. The computer program then estimated the numbers of sperm per seminal vesicle around 2000 in non-mated males and around 500 in males that were mated for two or three days. As our mated males showed a drastic reduction in sperm, we argue that we indeed have created a demand for sperm.

### Mating had no effect on GSC numbers

**Figure 3:**
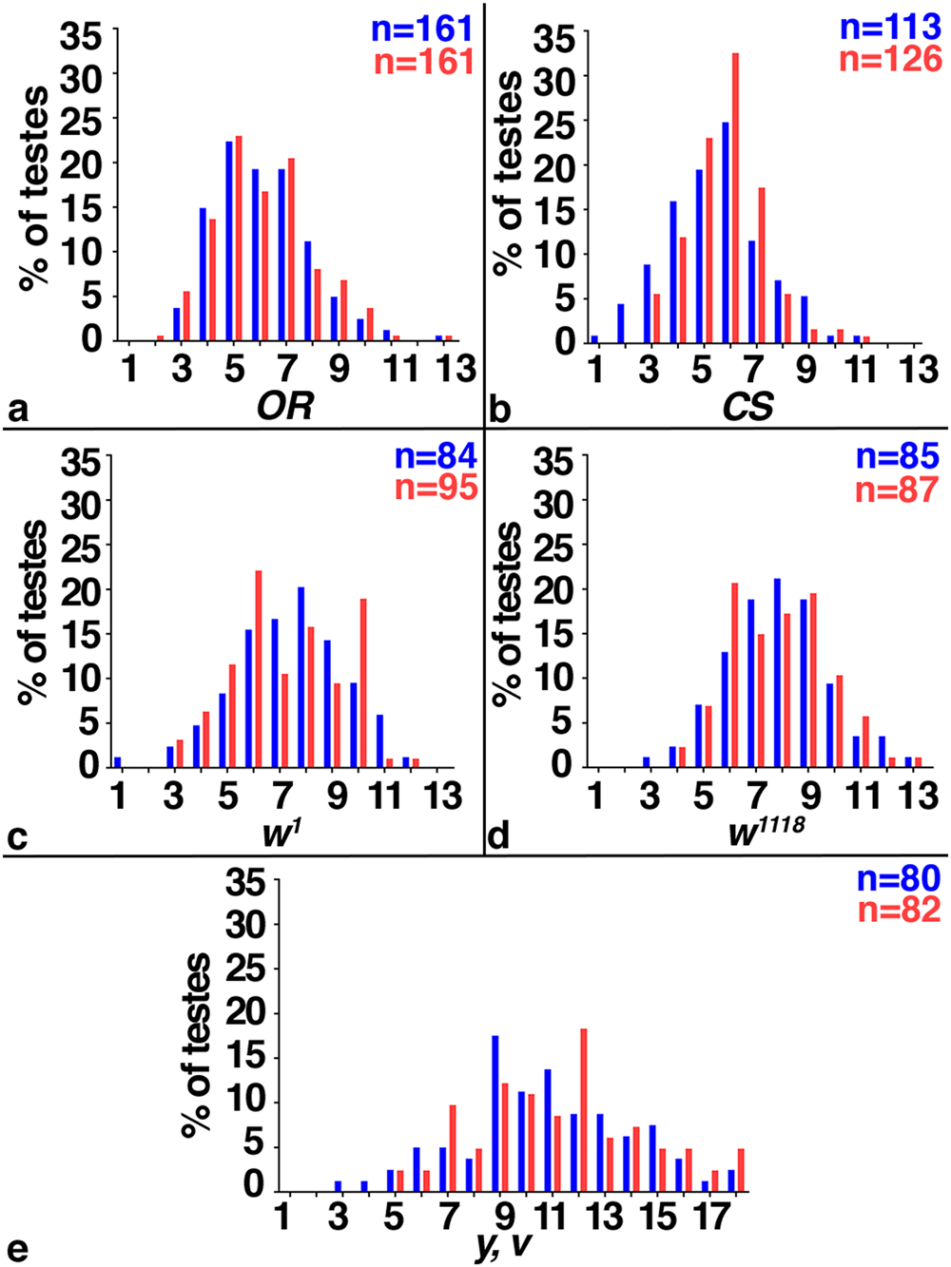
Mating did not affect GSC numbers. (**a–e**) FDGs showing numbers of GSCs on the X-axis and percentage of testes with the number of GSCs on the Y-axis. Blue: non-mated condition, red: mated condition, numbers of gonads (n=) and genotypes as indicated. No difference in GSC numbers was observed between non-mated and mated males from different genetic backgrounds.

It was previously reported that females significantly increased the numbers of their GSCs upon mating^34^. According to the literature, an adult male gonad contains up to twelve GSCs per testis, but the exact number of GSCs per testis appears to vary among different strains and laboratories. One study using a *wt* strain of males reported six to ten GSCs per testis, while other studies using transgenic males in a *w* mutant genetic background reported 8.94 and 12.3 GSCs per testis, respectively^36–38^. Among our fly lines, we found variation in GSC numbers as well. The distribution of GSCs ranged from one to 14 per testis, with an average of seven GSCs per testis. Males from an isogenized *OR* stock had the lowest average number of GSCs, having only four to five GSCs per testis (Fig. 3a). Males from an isogenized *CS* stock had an average of six GSCs per testis (Fig. 3b). Animals mutant for *w* alleles, *w*^1118^ and *w*^1^, had on average eight and seven GSCs per testis, respectively (Fig. 3c,d). Males from a *v*^1^*, y*^1^ stock, which serves as the genetic background for many RNA-*i* lines, had the highest average number of GSCs, at 11 GSCs per testis (Fig. 3e). We believe that the obtained GSC numbers are specific to the fly lines in our laboratory and do not necessarily reflect the numbers of GSCs in fly stocks of other laboratories.

Importantly, we did not observe a significant difference in the numbers of GSCs between non-mated and mated siblings in any of these fly lines (Fig. 3a–e). We conclude that mating did not affect the numbers of GSCs in our fly stocks. However, the observed variation in GSC numbers prompted us to perform our experiments in animals from as similar genetic backgrounds as possible. All males reported in the following of this manuscript carried the X-chromosome from our isogenized *OR* line.

### The increase in MI^GSC^ upon mating required G-protein signaling

**Figure 4:**
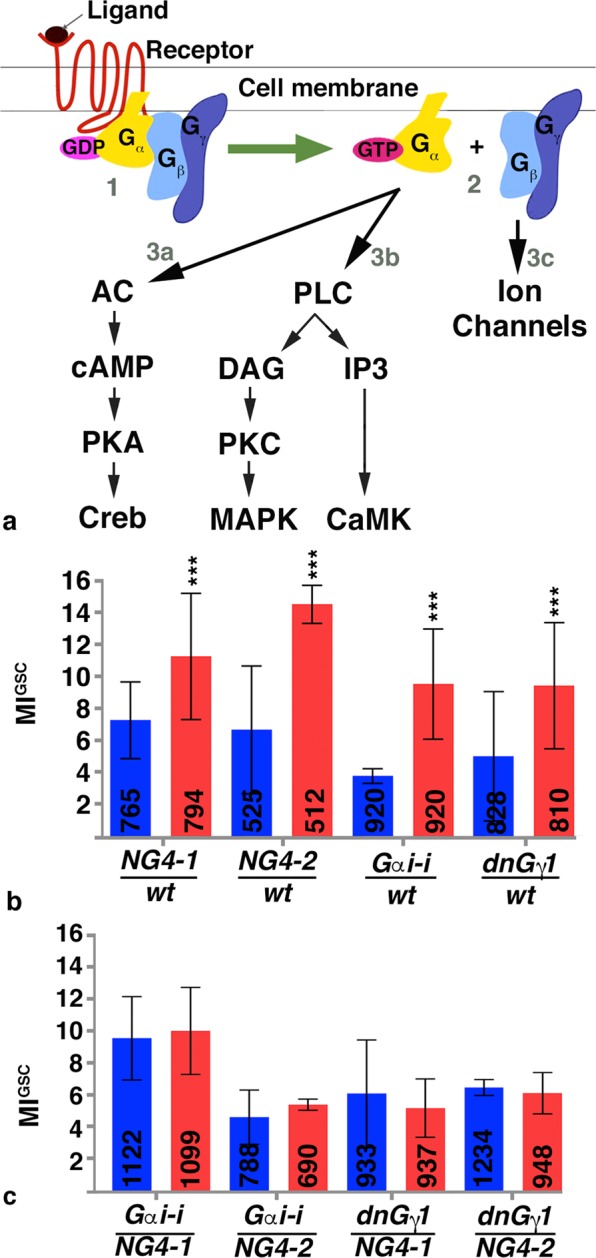
G-proteins were required for the increase in MI^GSC^ in response to mating. (**a**) Cartoon depicting the activation of G-proteins upon GPCR stimulation by ligand. 1: G-protein association before GPCR stimulation, 2: G-protein distribution after GPCR stimulation, 3a-c: downstream signaling cascades. AC: Adenylyl Cyclase, cAMP: cyclic Adenosine Monophosphate, PKA: Protein Kinase A, CREB: cAMP responsive element-binding protein, PLC: Phospho Lipase C, DAG: Diacylglycerol, PKC: Protein Kinase C, MAPK: Map Kinase, IP3: Inositol Triphosphate, CaMK: Calcium^2+/^calmodulin-dependent protein kinase. (**b,c**) Bar graphs showing MI^GSC^. Blue: non-mated condition, red: mated condition, ***P-value < 0.001, numbers of GSCs as indicated, genotypes as indicated. (**b**) Control animals increased MIs^GSC^ in response to mating. (**c**) Males expressing *G*_*α*_*i-i* or dn*G*_*γ*_1 in the germline did not increase MIs^GSC^ after mating.

*Drosophila* mating is a complex and genetically controlled behavior that is dependent on neural circuits^39^. This implicates a possible neuronal control in regulating GSC divisions during mating. Therefore, we wanted to focus on the type of signaling pathway commonly stimulated during neural activity, G-protein signaling^40,41^. In a non-stimulated cell, a trimeric complex of G-proteins, G_α_, G_β_, and G_γ_ is associated with classical GPCRs (Fig. 4a, step 1). When a ligand binds to the GPCR, a guanidyl exchange factor within the GPCR becomes activated that exchanges GDP for GTP in the G_α_ subunit. The exchange leads to the dissociation of G_α_ and the G_β/γ_ complex from each other and from the GPCR. Remaining attached to the membrane, G_α_ and G_β/γ_ diffuse along it and activate downstream signal transducers (Fig. 4a, step 2)^42,43^. Most organisms have multiple genes that encode each of the G-protein subunits. *Drosophila* has six G_α_, three G_β_, and two G_γ_ proteins, yet only a few examples are available in the literature associating a specific *Drosophila* G-protein with an upstream GPCR^42,44^.

Animals mutant for G-protein subunits are often lethal, making it problematic to investigate their roles in the adult. Furthermore, studying G-protein signaling in animals lacking their function throughout the whole body could affect behavior and physiology of the fly, leading to confounding effects on mating and GSC divisions. Fortunately, large collections of RNA-*i*-lines are available that are expressed under control of UAS. To reduce G-protein signaling we employed two separate *NG4*-lines, *NG4-*1 and *NG4-2*. When RNA-*i* against the different G-protein subunit was expressed via *NG4-1*, several of the mated males displayed only a weak increase in MI^GSC^ compared to their non-mated siblings (Table 1). We focused on an RNA-*i*-line that is directed against the subunit *G*_*α*_*i* as animals expressing this construct within the germline did not show any increase in MI^GSC^ in response to mating. For reproducibility, we conducted each of the following experiments in triplicates. We used progeny from transgenic Gal4 and UAS-flies that had been crossed to *wt* as positive controls. As expected, each population of positive control males displayed a significant increase in MI^GSC^ when mated (Fig. 4b). Experimental flies expressing *G*_*α*_*i-i* via *NG4-1* or *NG4-2*, however, failed to increase their MIs^GSC^ (Fig. 4c).

**Table 1:** MI^GSC^ from control, RNA-*i* and overexpression lines directed against G-protein subunits and other signal transducers.

Genotype	BL#	Crossed to:	MI^GSC^ Single	MI^GSC^ Mated	Diff.
*UAS-G*_*α*_*f-i*	43201	NG4-1	19/448 = 4.2%	52/452 = 11.5%	7.3
	25930*	NG4-1	54/1031 = 5.2%	76/1183 = 6.1%	0.9
*UAS-G*_*α*_*i-i*	34924	NG4-1	22/335 = 6.6%	49/322 = 15.2%	8.6
	40890	NG4-1	33/597 = 5.5%	59/536 = 11%	5.5
	31133	NG4-1	5/269 = 1.9%	19/285 = 6.7%	4.8
*UAS-G*_*α*_*o-i*	34653*	NG4-1	23/313 = 7.3%	26/295 = 8.8%	1.5
	28010	NG4-1	18/240 = 7.5%	27/231 = 11.7%	4.2
*UAS-G*_*α*_*q-i*	36820	NG4-1	24/403 = 6.0%	32/268 = 11.9%	5.9
	33765	NG4-1	33/320 = 10%	50/298 = 16.8%	6.8
	36775	NG4-1	9/153 = 5.9%	21/255 = 8.6%	2.7
	31268	OR	17/233 = 7.3	15/169 = 8.9	1.6
		OR	14/335 = 4.2	12/164 = 7.3	3.1
		OR	31/568 = 5.5	27/333 = 8.1	2.6
		NG4-1	25/556 = 4.5	8/296 = 2.7	−2.8
		NG4-1	23/332 = 6.9	23/291 = 7.9	1
		NG4-1	23/542 = 4.2	19/577 = 3.3	−0.9
		NG4-1	71/1430 = 5.0	50/1164 = 4.3	−0.7
	30735	NG4-1	14/318 = 4.4%	23/293 = 7.8%	3.4
*UAS-G*_*α*_*s-i*	29576	NG4-1	49/605 = 8.1%	60/615 = 9.7%	1.6
	50704	NG4-1	82/1137 = 7.3%	121/1527 = 7.9%	0.6
*UAS-G*_*β*_*5-i*	28310	NG4-1	10/306 = 3.3%	20/292 = 6.9%	3.6
*UAS-G*_*β*_*13F-i*	35041	NG4-1	38/752 = 4.8%	46/785 = 5.7%	0.9
	31134	NG4-1	12/198 = 6%	31/221 = 14%	8.0
*UAS-G*_*β*_*76C-i*	28507	NG4-1	14/219 = 6.4%	26/226 = 11.5%	5.1
*UAS-G*_*γ*_*1-i*	25934	NG4-1	21/283 = 7.4%	45/311 = 14.4%	7.0
	34372	NG4-1	21/434 = 4.8%	46/400 = 11.5%	6.7
*UAS-G*_*γ*_*30A-i*	25932	NG4-1	16/319 = 5.0%	18/286 = 6.3%	1.3
	34484	NG4-1	9/320 = 2.8%	31/323 = 9.6%	6.8
*UAS-CaMKI-i*	41900	NG4-1	17/337 = 5.0%	31/328 = 9.4%	4.4
	35362	NG4-1	10/222 = 4.5%	17/212 = 8.0%	3.5
	26726	NG4-1	16/301 = 5.3%	22/282 = 7.8%	2.5
*UAS-CaMKII-i*	35330	NG4-1	49/784 = 6.2%	91/858 = 10.6%	4.4
	29401	NG4-1	38/332 = 11.4%	43/342 = 12.6%	1.2
*UAS-CrebA-i*	42526	NG4-1	13/301 = 4.3%	29/298 = 9.7%	5.4
*UAS-Gprk1-i*	35246	NG4-1	13/323 = 4.0%	15/207 = 7.4%	3.4
	28354	NG4-1	13/304 = 4.3%	24/289 = 12%	7.7
*UAS-Gprk2-i*	41933	NG4-1	3/218 = 1.4%	24/228 = 10.5%	9.1
	35326	NG4-1	12/268 = 4.5%	35/267 = 13.1%	8.6
*UAS-IP3K-i*	35296	NG4-1	10/225 = 4.4%	14/152 = 9.2%	4.8
*UAS-PKC53E-i*	34716	NG4-1	18/304 = 5.9%	22/289 = 7.6%	1.7
*UAS-PKC98E-i*	29311	NG4-1	14/293 = 4.8%	15/284 = 5.3%	0.5
*UAS-PLC21C-i*	33719	NG4-1	23/264 = 8.7%	37/311 = 11.9%	3.2
*UAS-bsk-i*	53310	NG4-1	21/380 = 5.5%	32/346 = 9.3%	3.8
*UAS-Ira-i*	31595	NG4-1	6/272 = 2.2%	8/190 = 4.2%	2.0
*UAS-kay-i*	27722	NG4-1	11/256 = 4.3%	25/259 = 9.6%	5.3
	31322	NG4-1	22/384 = 5.7%	61/334 = 18.3%	12.6
	31391	NG4-1	24/291 = 8.2%	29/218-13.3%	5.1
*UAS-rl-i*	36059	NG4-1	21/269 = 7.8%	32/297 = 10.8%	3.0
*UAS-wt-5-HT1A*	27630	NG4-1	16/335 = 4.8%	30/302 = 10%	5.2
	27631	NG4-1	10/240 = 4.2%	24/271 = 8.9%	4.7
*UAS-wt-CrebB17A*	7220	NG4-1	51/636 = 8.0%	98/628 = 15.6%	7.6
	9232	NG4-1	32/637 = 5.0%	84/592 = 14.2%	9.2
*UAS-wt-CaMK2R3*	29662	NG4-1	20/292 = 6.8%	28/289 = 10.0%	3.2
*UAS-CaMKII.T287A/*	29663	NG4-1	16/287 = 5.6%	25/283 = 8.8%	3.2
*UAS-wt-Gαs*	6489	NG4-1	32/289 = 11.1%	39/300 = 13.0%	1.9
	6489	NG4-1	40/610 = 6.6%	54/652 = 8.3%	1.7
*UAS-wt-Ira*	7216	NG4-1	15/342 = 4.4%	44/378 = 11.6%	7.2
*UAS-wt-Kay*	7213	NG4-1	24/341 = 7.4%	70/350 = 20.0%	12.6

UAS-driven expression for the listed genes in the germline via *NG4-1*. BL #: Bloomington stock number, Single and Mated: number of pHH3-positive GSCs/total number of GSCs = MI^GSC^, Diff: MI^GSC^ of mated males minus MI^GSC^ of non-mated males. Note the variability in MI^GSC^ among the different genotypes. For RNA-*i*-lines marked by asterisks siblings outcrossed to *wt* did not show a strong response to mating either, suggesting leakiness of the lines.

We next sought to validate the role for G-protein signaling in GSC division frequency by an alternative approach. A dominant negative version of *Drosophila* G_γ_1 (dnG_γ_1) is available that serves as a reliable tool to abolish G-protein signaling^45^. Males expressing dnG_γ_1 via either *NG4-1* or *NG4-2* did not show an increase in MI^GSC^ in response to mating (Fig. 4c). Control dnG_γ_1/*wt* animals, on the other hand, had an increased MI^GSC^ upon mating (Fig. 4b). These data clearly show that signaling via G-proteins is required for the increase in MI^GSC^. Plotting the results in FDGs confirmed that mated control animals had significantly fewer testes with an MI^GSC^ of zero and more testes with a higher MI^GSC^ compared to non-mated males (Supplemental Fig. S3a–d), and that this response to mating was eliminated in experimental males (Supplemental Fig. S3e–h).

In mammalian cells, three major G-protein-dependent signaling cascades have been described (Fig. 4a, steps 3a, b, c)^40,46^. For *Drosophila*, the literature provides little information on the signaling cascades downstream of GPCRs but it is generally assumed that the mammalian signal transducers are conserved in flies. To validate that an increase in MI^GSC^ upon mating is regulated by G-protein signaling we expressed RNA-*i* and mis-expression constructs for conserved signal transducers via NG4 and found that males expressing RNA-*i*-lines for one of the *Drosophila Protein Kinase C* (*PKC*) proteins, *PKC98E*, and for *Inositol-triphosphate 3-Kinase* (*IP3K)* indeed failed to increase MIs^GSC^ in response to mating (Supplemental Fig. S2c).

### RNA-*i* against seven distinct GPCRs blocked the increase in MI^GSC^ upon mating

**Figure 5:**
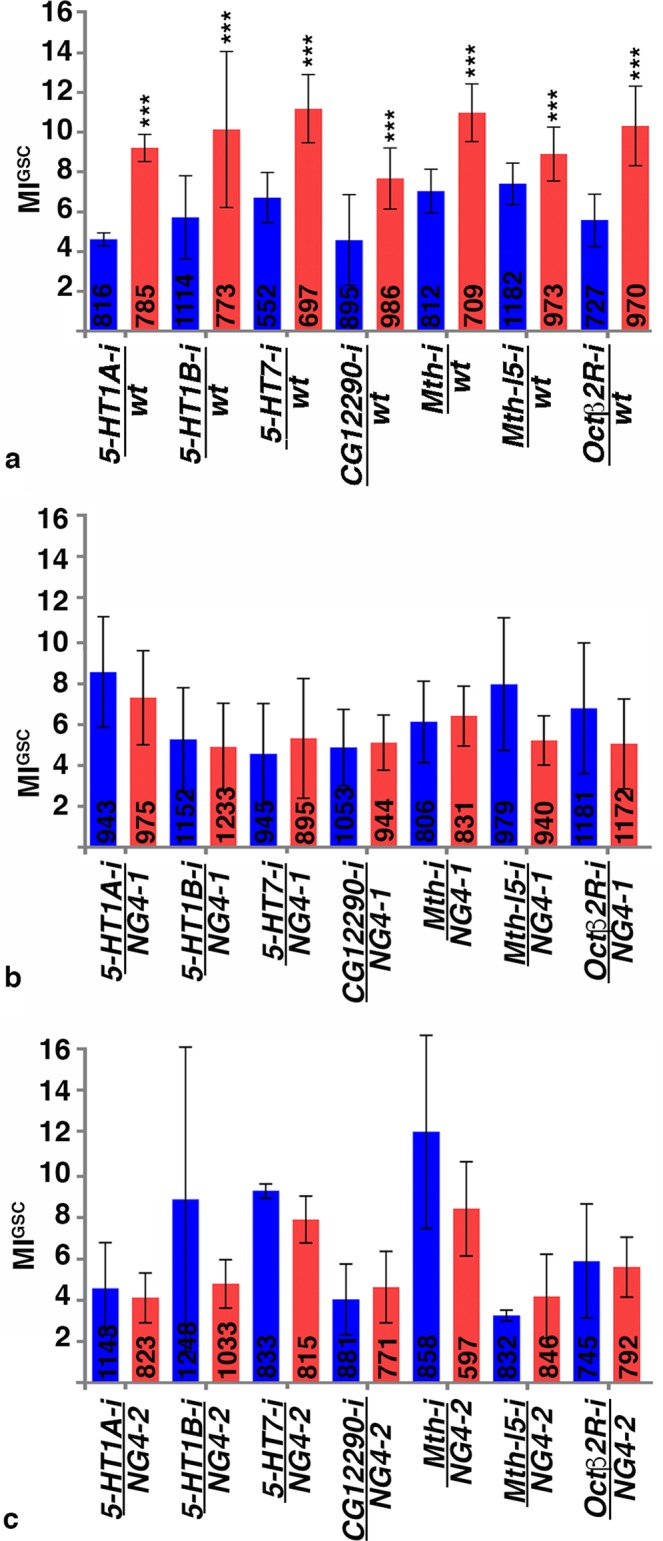
Expression of RNA-*i* against seven distinct GPCRs blocked the increase in MI^GSC^ in response to mating. (**a–c**) Bar graphs showing MI^GSC^. Blue: non-mated condition, red: mated condition, ***P-value <0.001, numbers of GSCs as indicated, genotypes as indicated. (**a**) Control males have significantly higher MIs^GSC^ than their non-mated siblings. (**b,c**) Mated (**b**) *GPCR-i*/NG4-1 and (**c**) *GPCR-i*/NG4-2 males did not have significantly higher MIs^GSC^ compared to their non-mated siblings.

To further confirm that G-protein signaling regulates the increase in MI^GSC^ we aimed towards identifying the upstream GPCRs. Next Generation Sequencing (NGS) of RNA from *wt* testis tips revealed the expression of 143 peptide, hormone, and growth factor receptors, including 35 classical GPCRs (Supplemental Table S1). The functions of many of these GPCRs have not been studied yet and mutant animals are only available in rare cases. Expressing RNA-*i*-constructs against most GPCRs in the germline had little to no effect on the ability of the GSCs to increase their MI^GSC^ in response to mating (Table 2). RNA-*i* against three Serotonin Receptors (5-HT1A, 5-HT1B and 5-HT7), Mth, Mth-l5, Octβ2R, and a predicted GPCR encoded by CG12290, clearly and reproducibly eliminated this ability. Animals carrying UAS-controlled RNA-*i*-constructs against these GPCRs (*GPCR-i*) were crossed to *wt*, *NG4-1* and *NG4-2*, and MI^GSC^ of their progeny was investigated. Each of the controls (*GPCR-i/wt*) increased their MI^GSC^ when repeatedly mated to females in each of the triplicate experiments (Figs. 5a and S4a–g). However, when the *GPCR-i*-animals were crossed with either *NG4-1* (Fig. 5b and Supplemental Fig. S4h–n) or *NG4-2* (Fig. 5c and Supplemental Fig. S4o–u) the MIs^GSC^ of their non-mated and mated progeny did not significantly differ. Confirming the necessity of the GPCRs in increasing MI^GSC^, we investigated alternative RNA-*i*-lines. A second RNA-*i*-line for Mth blocked the increase in MI^GSC^ in mated males and a second RNA-*i*-line for 5-HT1A displayed only a weak response to mating (Table 3). Viable alleles of 5-HT1A and 5-HT1B were not pursued as alternative strategies because they displayed only a weak mating success rate (Supplemental Table S2).

**Table 2:** MI^GSC^ from select RNA-*i*-lines directed against GPCRs.

GPCR	BL #	Single	Mated	Diff.
*UAS-5-HT2A-i*	31882	25/490 = 5.1	36/465 = 7.3	2.2
	56870	19/582 = 3.3	32/553 = 5.8	2.5
*UAS-5-HT2B-i*	60488	6/261 = 2.3	21/272 = 7.7	5.4
	25874	4/228 = 1.7	34/236 = 14.4	12.7
*UAS-Ado-R-i*	27536	11/276 = 4.0	20/209 = 9.6	5.6
*UAS-AKHR-i*	29577	23/492 = 4.7	54/574 = 9.4	4.7
*UAS-AR-2-i*	25935	13/363 = 3.6	25/336 = 7.4	3.6
*UAS-CG13229-i*	29519	9/285 = 3.2	33/297 = 11.1	7.9
*UAS-CG14539-i*	25855	21/318 = 6.6	31/307 = 10.1	3.5
*UAS-CG15556-i*	44574	28/425 = 6.6	43/401 = 10.7	4.1
*UAS-CG15744-i*	28516	18/279 = 6.4	27/252 = 10.7	4.3
	42497	23/323 = 7.1	36/236 = 11.0	3.9
*UAS-CG30106-i*	27669	10/244 = 4.1	34/273 = 12.4	8.3
*UAS-CG33639-i*	28614	32/300 = 10.7	47/371 = 12.7	2.0
UAS-CCHaR1-i*	51168	27/407 = 6.6	27/323 = 8.4	1.8
*UAS-Cry-i*	43217	43/389 = 11.0	75/521 = 14.4	3.4
*UAS-CrzR-i*	52751	14/337 = 4.2	31/333 = 9.3	5.1
*UAS-Dop1R1-i*	62193	12/352 = 3.4	25/308 = 8.1	4.6
	55239	11/300 = 3.7	44/267 = 16.5	12.8
*UAS-GABA BR2-i*	50608	6/176 = 3.4	35/207 = 16.9	13.5
	27699	7/291 = 2.4	18/282 = 6.4	4.0
*UAS-GABA BR3-i*	42725	10/190 = 5.3	28/243 = 11.5	6.2
*UAS-Moody-i*	36821	5/301 = 1.7	16/234 = 6.8	5.1
*UAS-Mth-l1-i*	41930	11/279 = 4.0	42/300 = 14.0	10
*UAS-Mth-l3-i*	41877	54/817 = 6.6	81/850 = 11.8	5.2
	36822	19/231 = 8.2	39/241 = 16.2	8.0
*UAS-Mth-l8-i*	36886	48/933 = 5.1	70/915 = 7.6	2.5
*UAS-Mth-l9-i*	51695	61/985 = 6.2	82/910 = 9.0	2.8
*UAS-Mth-l15-i*	28017	14/349 = 4.0	25/337 = 7.1	3.1
*UAS-PK1R-i*	27539	28/478 = 5.9	37/375 = 9.9	4.0
*UAS-Smo-i*	27037	12/263 = 4.6	33/288 = 11.4	6.8
	43134	19/274 = 7.0	15/147 = 10.2	3.2
*UAS-Tre1-i*	34956	5/234 = 2.1	28/252 = 11.1	9.0
UAS-TKR86D-i*	31884	31/564 = 5.5	28/394 = 7.1	1.6
*UAS-TKR99D-i*	55732	30/506 = 5.9	49/467 = 10.5	4.6
	27513	4/240 = 1.7	30/294 = 10.2	8.5

UAS-driven expression of RNA-*i* for the listed GPCRs via *NG4-1* did not block the increase in MI^GSC^ in response to mating. BL #: Bloomington stock number, Single and Mated: number of pHH3-positive GSCs/total number of GSCs = MI^GSC^, Diff: MI^GSC^ of mated males minus MI^GSC^ of non-mated males. GPCRs marked by asterisks were excluded from further studies because their siblings outcrossed to *wt* did not show a stronger response to mating than the experimental (GPCR-*i*/*NG4-1*) flies.

**Table 3:** MI^GSC^ from additional RNA*i*-lines directed against the GPCRs blocking the increase in MI^GSC^ in mated males.

GPCR	BL #	Single	Mated	Diff.
*5-HT1A-i/NG4-1*	25834	64/841 = 7.6	67/777 = 8.6	1
*5-HT1B-i/NG4-1*	25833	16/256 = 6.2	26/268 = 9.7	3.5
	27635	3/304 = 1.0	27/317 = 8.5	7.5
	51842	20/381 = 5.2	32/375-8.5	3.3
	54006	13/405 = 3.2	23/351 = 6.5	3.3
*5-HT7-i/NG4-1*	32471	8/238 = 3.4	17/229 = 7.4	4.0
*CG12290-i/NG4-1*	42520	4/260 = 1.5	31/246 = 12.6	11.1
*Mth-i/NG4-1*	27495	14/352 = 4	15/336 = 4.5	0.5

BL #: Bloomington stock number, Single and Mated: number of pHH3-positive GSCs/total number of GSCs = MI^GSC^, Diff: MI^GSC^ of mated males minus MI^GSC^ of non-mated males.

To assure that the males actually mated, we used two criteria: visual observation and the appearance of progeny. When flies were anesthetized to exchange the females for fresh virgins, several copulating pairs of males and females were always observed. Furthermore, 100 single females that had been exposed to males on day one of the experiment were placed into one food vial each and mating success evaluated a few days later by counting the percentage of vials with progeny. Most males in this study sired 60–90% of the females. Specifically, each of the *GPCR-i/NG4-1* males produced offspring (Supplemental Table S2), showing that a block in the increase in MI^GSC^ was not caused by a failure to mate but by expressing RNA-*i* directed against the GPCRs.

Finally, we wanted to assure that the age of the males had no effect on the increase in MI^GSC^. We performed a time-course experiment of one, two, three, and four-week old *OR* males. We found that mated males of all ages showed robust increases in MI^GSC^ compared to their non-mated siblings (Supplemental Fig. S2d). We conclude that aging animals up to four weeks had little to no effect on the ability of *wt* GSCs to increase their MI^GSC^ in response to mating, and that the age of the transgenic animals used in this study (three weeks of age at the time of testes dissection) had no impact on the obtained results.

## Discussion

Here, we show that repeated mating reduced the sperm pool and increased GSC division frequency. Using highly controlled experiments, we demonstrate that mated males had more GSCs in M-phase and S-phase of the cell cycle compared to non-mated males. Mated males also showed faster incorporation of EdU in feeding experiments, suggesting that the GSCs of mated males entered the S-phase of the cell cycle more frequently. Though we cannot exclude the possibility that mated males ingested more EdU-supplemented food than their non-mated siblings, our data suggest that the GSCs in mated males cycle faster. The response curve we obtained in our time-course experiment is different from the response curves reported by other groups that used bromo-deoxy-uridine (BrDU) as the thymidine analog instead of EdU. For example, the non-mated males in our experiment had about 70% of EdU-positive GSCs after 48 hours of feeding. A study that uses *white* (*w*) mutant animals and fed the same concentration of the thymidine homologue had a steeper response curve, in which 85% of the GSCs were BrDU-marked after 48 hours of feeding^30^. Another study using *y, v* flies showed even steeper response curves where 100% of the GSCs were BrDU-labeled after 24 hours. However, in this study, animals were fed a 30 times higher concentration of the thymidine homologue than used in our study^47^. We propose that the different response curves are due to the different genetic backgrounds, chemicals, and doses.

Our findings demonstrate that GSCs can respond to a demand for sperm by increasing their mitotic activity. Based on RNA-*i* targeting G-proteins and a dominant negative construct against G_γ_1, the increase in MI^GSC^ of mated males is dependent on G-protein signaling. Furthermore, signal transducers predicted to act downstream of G-proteins and GPCRs predicted to act upstream of G-proteins also appeared to be required for the response to mating. Whether G-protein signaling directly affects GSC division frequency, or whether G-protein-dependent communication among the early stage germline cells impacts the MI^GSC^ remains to be investigated.

Due to the lack of mutants and a potential interference of whole animal knock-down in the behavior of the flies, we used tissue-specific expression of RNA-*i*-constructs. It is surprising that our studies revealed potential roles for seven instead of a single GPCR in the increase of MI^GSC^ in response to mating. A possible explanation is that some of the RNA-*i*-lines have off-target effects. RNA*-i*-hairpins can cause the down-regulation of unintended targets due to stretches of sequence homologies, especially when long hairpins are used^48,49^. However, with the exception of the RNA-*i*-line directed against *5-HT7*, all lines that produced a phenotype contain second generation vectors with a short, 21 nucleotide hairpin predicted to have no off-target effects^50^. Thus, we hypothesize that multiple GPCRs regulate the increase in MI^GSC^ in response to mating. Consistent with this, expression of other RNA-*i*-lines directed against *Mth* or *5-HT1A* interfered with the increase in MI^GSC^ in mated males.

Our finding that RNA-*i* against several GPCRs blocked the increase in MI^GSC^ in mated males suggests a high level of complexity in the regulation of GSC division frequency. One simple explanation could be that the increase in GSC division frequency is dependent on ideal physiological conditions and that lack of any of the seven GPCRs somehow impairs the cell’s normal metabolism. Alternatively, the GPCRs could act in concert to impact the MI^GSC^. In the literature, increasing evidence has emerged that GPCRs can form dimers and oligomers and that these have a variety of functional roles, ranging from GPCR trafficking to modification of G-protein mediated signaling^51–53^. In *C. elegans*, two Octopamine receptors, SER-3 and SER-6, additively regulate the same signal transducers for food-deprived-mediated signaling. One possible explanation for the non-redundant function of the two receptors was the idea that they form a functional dimer^54^. In mammalian cells, 5-HT receptors can form homo-dimers and hetero-dimers and, dependent on this, have different effects on G-protein signaling^55–57^. In cultured fibroblast cells, for example, G-protein coupling is more efficient when both receptors within a 5-HT4 homo-dimer bind to agonist instead of only one^58^. In cultured hippocampal neurons, hetero-dimerization of 5-HT1A with 5-HT7 reduces G-protein activation and decreases the opening of a potassium channel compared to 5-HT1A homo-dimers^59^. The formation of hetero-dimers of GPCRs with other types of receptors plays a role in depression and in the response to hallucinogens in rodents^60,61^.

Alternatively, or in addition to the possibility that some or all of the seven GPCRs form physical complexes, a role for several distinct GPCRs in regulating GSC division frequency could be explained by cross-talk among the downstream signaling cascades. One signaling cascade could, for example, lead to the expression of a kinase that is activated by another cascade. Similarly, one signaling cascade could open an ion channel necessary for the activity of a protein within another cascade. Unfortunately, the literature provides little information on *Drosophila* GPCR signal transduction cascades and only very few mutants have been identified that affect a process downstream of GPCR stimulation. Thus, it remains to be explored how stimulation of the GPCRs and G-proteins increase GSC divisions.

The role for G-protein signaling in regulating the frequency of GSC divisions is novel. Our data suggest that the increase in MI^GSC^ in response to mating is regulated by external signals, potentially arising from the nervous system, that stimulate G-protein signaling in the germline. Based on the nature of the GPCRs, the activating signal could be Serotonin, the Mth ligand, Stunted, Octopamine, or two other, yet unknown, signals that activate Mth-l5, and CG12290^62–64^. It will be interesting to address which of these ligands are sufficient to increase MI^GSC^, in what concentrations they act, by which tissues they are released, and whether they also affect other stem cell populations.

## Methods

### Fly husbandry

Flies were raised on a standard cornmeal/agar diet and maintained in temperature-, light-, and humidity-controlled incubators. All mutations, markers, and transgenic lines are described in the *Drosophila* database. Unless otherwise noted, flies were obtained from the Bloomington stock center^65^. The *NG4-1*-line is of unknown origin; *NG4-2*: BL#4937; *G*_*α*_*i-i:* BL#35407*; dnG*_*γ*_*1*: BL: 44604; *5-HT1A-i*: BL#33885; *5-HT1B-i*: BL#33418; *5-HT7-i*: BL#27272; *Mth-i*: BL#36823; *Mth-l5 i*: BL#36884; *Octβ-2R-i*: BL#50580; *CG12290-i*: BL#31873. All other Bloomington stock numbers are provided in the tables and figures.

### UAS/Gal4-expression studies

X; UAS-*dicer*; *NG4* or *OR* females were crossed with males carrying target genes under the control of UAS in egg lay containers with fresh apple juice-agar and yeast paste to generate either experimental or control flies. The progeny were transferred into food bottles, raised to adulthood at 18 °C, males collected, and then shifted to 29 °C for seven days to induce high activity of Gal4 prior to the mating experiment. Note that the males were not collected as virgins as to avoid any potential developmental or learning effects on our experiments.

### Mating experiments

Unless otherwise noted, mating experiments were performed at 29 °C. Males and virgin females were placed in separate egg lay containers with apple juice-agar plates/yeast paste overnight to assure they were well fed prior to their transfer into mating chambers. Single males were placed into each mating slot either by themselves (non-mated) or with three virgin females (mated) and the chambers closed with apple juice-agar lids with yeast paste. Females were replaced by virgin females on each of the following two days and apple juice-agar lids with yeast paste were replaced on a daily basis for both non-mated and mated animals. In most instances, females from the stock *X*^∧^*X*, *y, w, f* / Y / *shi*^*ts*^ were used as virgins. When raised at 29 °C, only females hatch from this stock. For fertility tests, *OR* virgins were used. Note that 10–20% of the mated males died during a simple mating experiment while only 5% of the non-mated siblings died.

### Cuticular hormone extracts, net separation, and decapitation experiments

Flies were sorted by gender and soaked in hexanes for 30 minutes, vortexed for 2 minutes, and centrifuged for 1 minute as previously described^22^. The cuticular hormone containing supernatant was mixed into yeast paste and soaked onto apple juice-agar plates. Males were exposed to a fresh dose each day on three subsequent days.

For net separation experiments, animals were placed in groups of 50 into either side of a feeding construction in which two chambers were separated by nets and each chamber was closed with an apple-juice-agar plate with yeast. The plates were replaced every day with fresh plates every day for three days.

Females were decapitated with razor blades and kept on humid paper towels while exposed to males. The females were replaced every day for three days.

### Courtship experiments

*fru* mutant males were placed in egg lay containers with apple juice-agar plates/yeast paste overnight to assure they were well fed. Single males were then placed into mating chambers as controls, while 100 males were housed together in egg lay containers. Apple juice plates were replaced on each of the three days.

### Immunofluorescence and microscopy

Animals were placed on ice to immobilize them. Gonads were dissected in Tissue Isolation Buffer (TIB) and collected in a 1.5 ml tube with TIB buffer on ice for no more than 30 minutes. Gonads were then fixed, followed by immunofluorescence staining and imaging as previously described^7^. The mouse anti-FasciclinIII (FasIII) antibody (1:10) developed by C. Goodman was obtained from the Developmental Studies Hybridoma Bank, created by the NICHD of the NIH and maintained at The University of Iowa, Department of Biology, Iowa City, IA 52242. Goat anti-Vasa antibody (1:50 to 1:500) was obtained from Santa Cruz Biotechnology Inc. (sc26877), anti-phosphorylated Histone H3 (pHH3) antibodies (1:100 to 1:1000) were obtained from Fisher (PA5-17869), Milllipore (06-570), and Santa Cruz Biotechnology Inc. (sc8656-R). Secondary Alexa 488, 568, and 647-coupled antibodies (1:1000) and Slow Fade Gold embedding medium with DAPI were obtained from Life Technologies. Images were taken with a Zeiss Axiophot, equipped with a digital camera, an apotome, and Axiovision Rel. software.

### EdU-labeling experiments

The EdU-labeling kit was obtained from Invitrogen and the procedure performed following manufacturer’s instructions. For EdU-pulse labeling experiments, animals were mated as described above, and the dissected testes incubated with 10 mM EdU in PBS for 30 minutes at room temperature prior to fixation. For EdU-feeding experiments, *OR* males were fed 10 mM EdU in liquid yeast provided on paper towels. These animals were mated at room temperature (21 °C) because the paper towels easily dried out at higher temperatures, causing the flies to dehydrate and die.

### Graphic presentations and data analysis

All bar graphs, box plots, and FDGs were generated using GraphPad prism version 7 and incorporated into composite images using Adobe Photoshop. Statistical relevance was analyzed using the GraphPad prism default two-tailed student’s t-test, with the exception of the ImageJ data, for which we used the unpaired t-test.

To analyze the size of the seminal vesicles, z-stacks were flattened using the maximum intensity z-projection tool of the Axiovision Rel. program. The sizes of the seminal vesicle were first evaluated using the scale bar function of Axiovision Rel. Seminal vesicles broader than 120 μm were classified as wide (class 1) and those of or below 100 μm were classified as thin (class 2 and 3). Select images were then opened in ImageJ and the relevant area masked. Using the area function of ImageJ, we calculated the GFP-occupied area in square microns.

### Sperm head volumetric calculations

In order to evaluate sperm numbers, we turned to computer analysis in Python, which first quantified the volume of GFP signal occupied by all sperm heads. From there, it generated estimates to the amount of sperm in each seminal vesicle, based on the volume a single sperm head occupies. For the procedure, image stacks of individual seminal vesicles were opened. Next, relevant regions were masked. Then, we used the functions of the program to adjust the images. Each image set was normalized by mean subtraction and division by the standard deviation. Image intensity was rescaled to encompass the range of the image. To remove signal noise, a median filter was applied and the mask refined by Otso thresholding. We determined signal volume by hysteresis thresholding. This approach initially thresholds an image at an upper limit, and then expands the region by adjacent pixels satisfying the lower threshold. We set the lower boundary at the value generated from a triangle threshold. A triangle threshold assumes a unimodal distribution of pixels and takes a line from the most frequent intensity value to the highest intensity value on a histogram. It then determines the furthest perpendicular point from that line as the threshold. The upper threshold was then set as the median intensity value of a histogram consisting of the remaining values above the triangle threshold. The number of signal voxels was calculated and normalized to an expected size of a single sperm head. Our analysis utilized OpenCV 3.4.2, Scipy1.2.1, Scikit-image 0.14.2, Numpy 1.16.2, Matplotlib 3.0.3, Seaborn 0.9.0, as well as built in Python 3.7.3 modules^66–70^.

### Generation of RNA-profiles

Testes tips were cut in TIB using surgical needles and immediately transferred into Trizol reagent. Total RNA was extracted using the Direct-zol RNA MiniPrep kit from Zymo Research and following the manufacturer’s instructions. The quality of total RNA was assayed by microcapillary electrophoresis with a Biorad BioAnalyzer. Three replicates were then used for linear amplification, library construction, and sequencing (paired-end 100 bp (PE100) run on the Illumina HiSeq2000 platform) by the Georgia Genomics Facility. Resulting sequences were analyzed using TopHat and Cuffdiff2^71,72^.

## Supplementary information

Supplemental Data.

## Data Availability

The RNA-sequencing datasets generated and analyzed in this study are available from the authors upon request and will also be deposited through open source platforms.
