# Supplementary material for: G-protein signaling is required for increasing germline stem cell division frequency in response to mating in Drosophila males
Source: Sci Rep. 2020 Mar 3;10:3888. doi: 10.1038/s41598-020-60807-8 (PMC7054589; doi:10.1038/s41598-020-60807-8)
Supplement: Supplementary file 1 — Supplemental Data. [file 41598_2020_60807_MOESM1_ESM.docx]

**
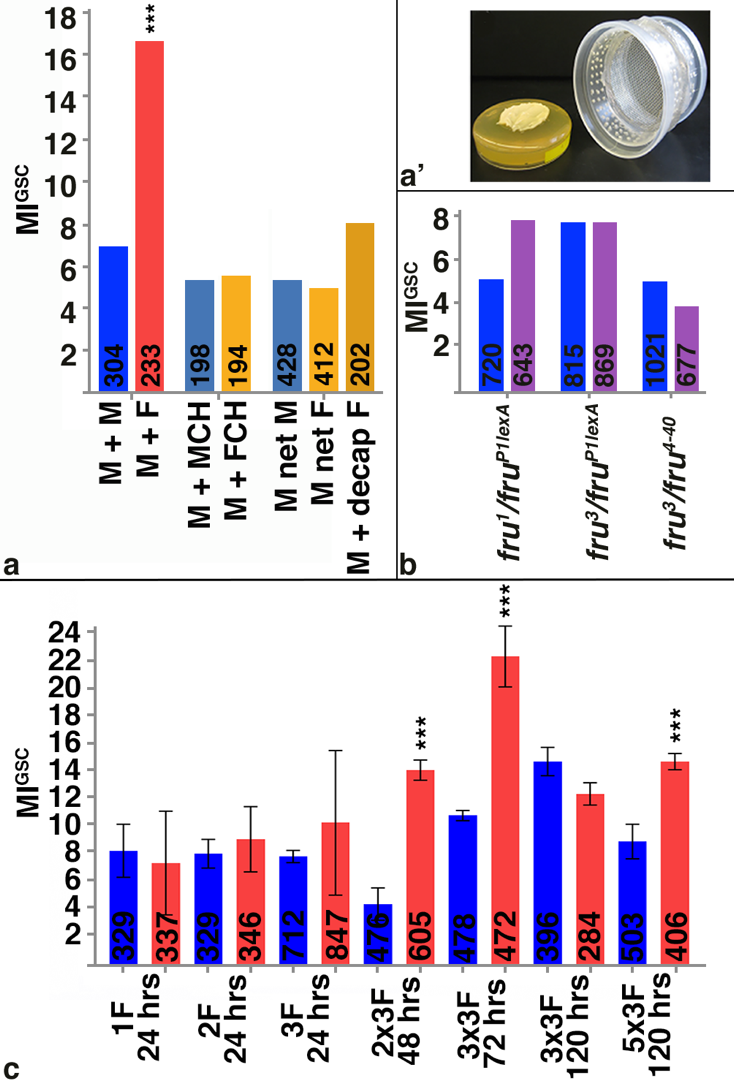
**

**Fig. S1. Validation of mating conditions for the increase in MI^GSC^.**

a-c) Bar graphs showing MIs^GSC^. ***: P-value < 0.001, numbers of GSCs and genotypes as indicated.

a) Mating, but not exposure to visual and chemical cues increased MI^GSC^ after three days of treatment. M: males; F: female virgins, CH: cuticular hormones, net: net separated, decap: decapitated.

a’) Feeding construction used to separate flies.

b) No difference in MIs^GSC^ were seen in *fru* mutant males kept either by themselves (blue bars) or exposed to each other (purple bars).

c) MIs^GSC^ after different mating conditions, as indicated. Blue: non-mated condition, red: mated condition, F: female virgins, hrs: hours. Blue: non-mated condition, red: mated condition.


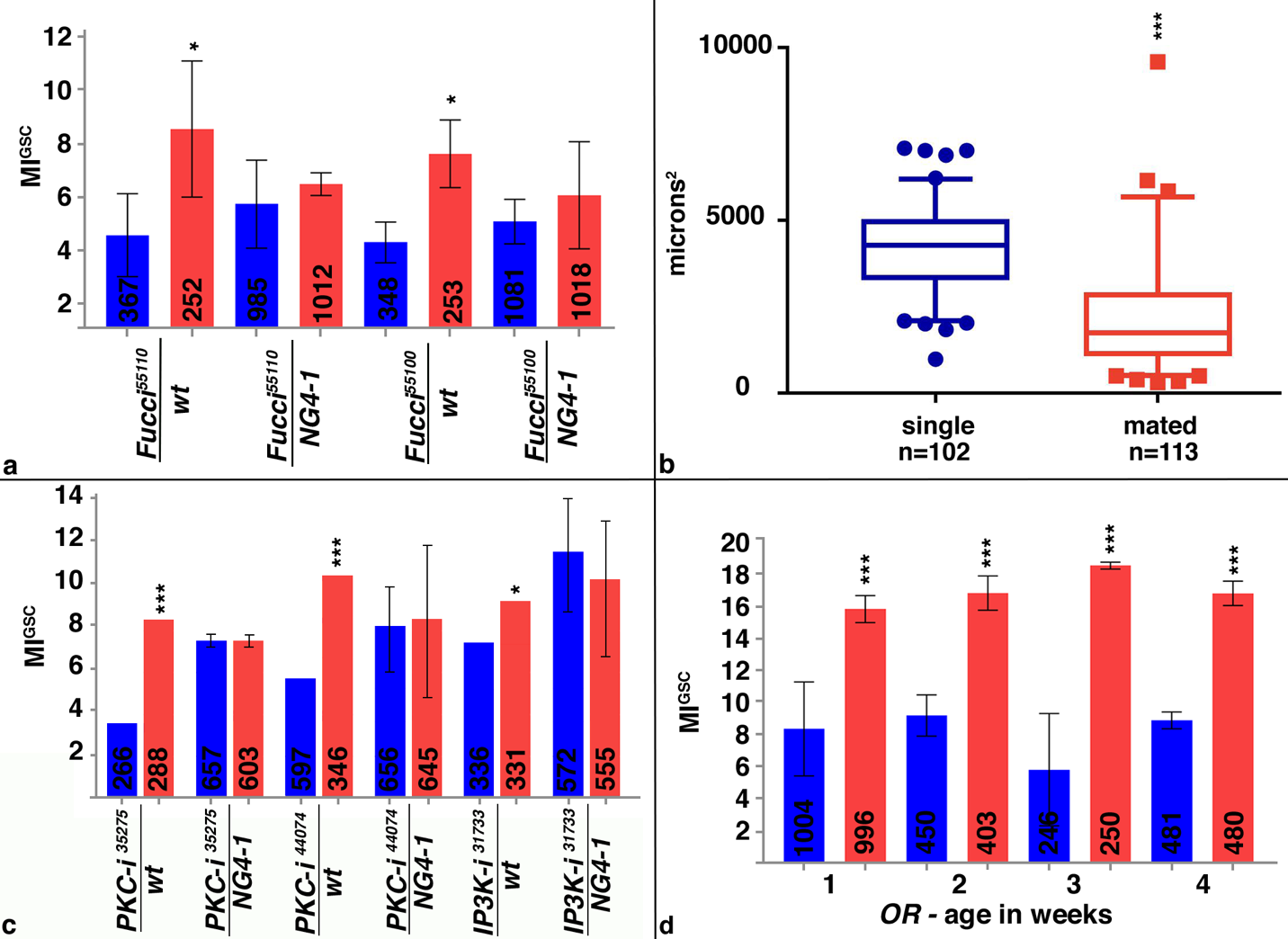


**Fig. S2. The impact of mating on MI^GSC^ and seminal vesicle size in various genetic backgrounds.**

a-d) Blue: non-mated condition, red: mated condition, ***: P-value < 0.001, *: P-value < 0.05, numbers of GSCs and genotypes as indicated, .

a) Bar graph showing MIs^GSC^ from experimental (*Fucci/NG4*) and control (*Fucci/wt*) Fucci-lines.

b) Box plot showing that mated males had significantly smaller seminal vesicles compared to their non-mated siblings, lines in the boxes represent medians, whiskers represent outlies, numbers of seminal vesicles (n=) as indicated.

c) Bar graph showing MIs^GSC^ from flies expressing RNA-*i* directed against PKC98C and IP3K. Mated experimental males (*RNA-i/NG4*) failed to increase MI^GSC^, while mated control males (*RNA-i/wt*) did not.

d) Bar graph showing MIs^GSC^ of non-mated and mated *OR* males at one, two, three, and four weeks of age.

**
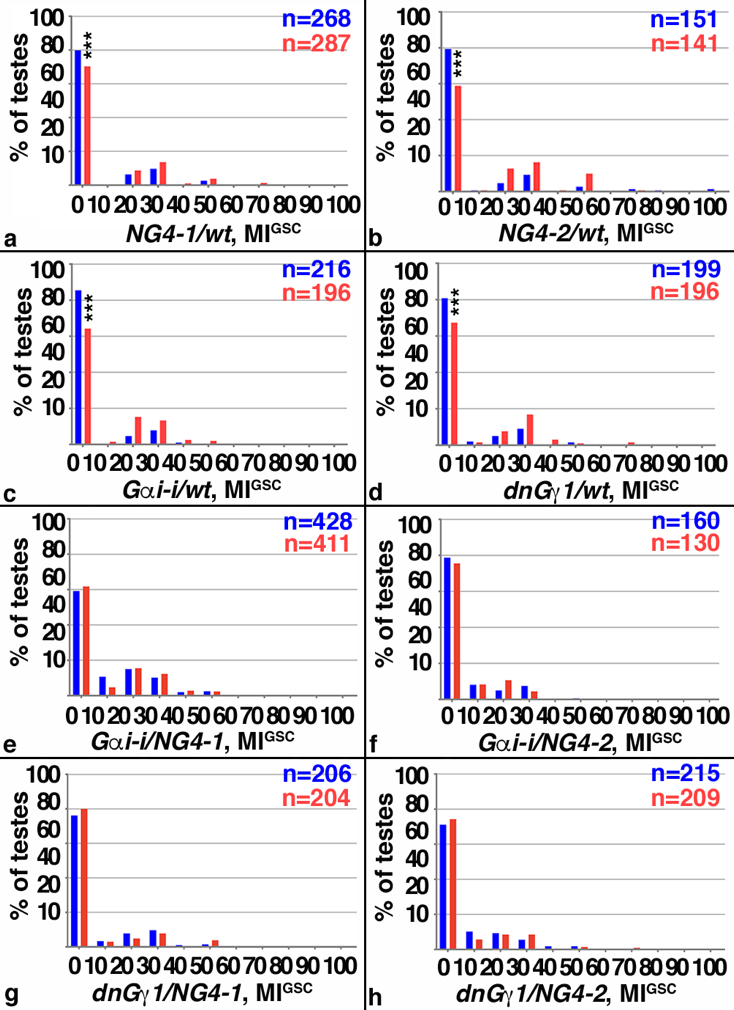
**

**Fig. S3. Modulated G-protein expression did not significantly change the distribution of MI^GSC^ across the population of testes.**

a-h) FDGs showing median of bin of MI^GSC^ across populations of males on the X-axis (bin width=10) and the percentage of testes with each MI^GSC^ on the Y-axis. Blue: non-mated condition, red: mated condition, n: number of testes examined, genotypes as indicated, ***: P-value < 0.001.

a-d) Control males

e-h) Males expressing *G_α_i-i* or dnG_γ_1 in the germline.

**
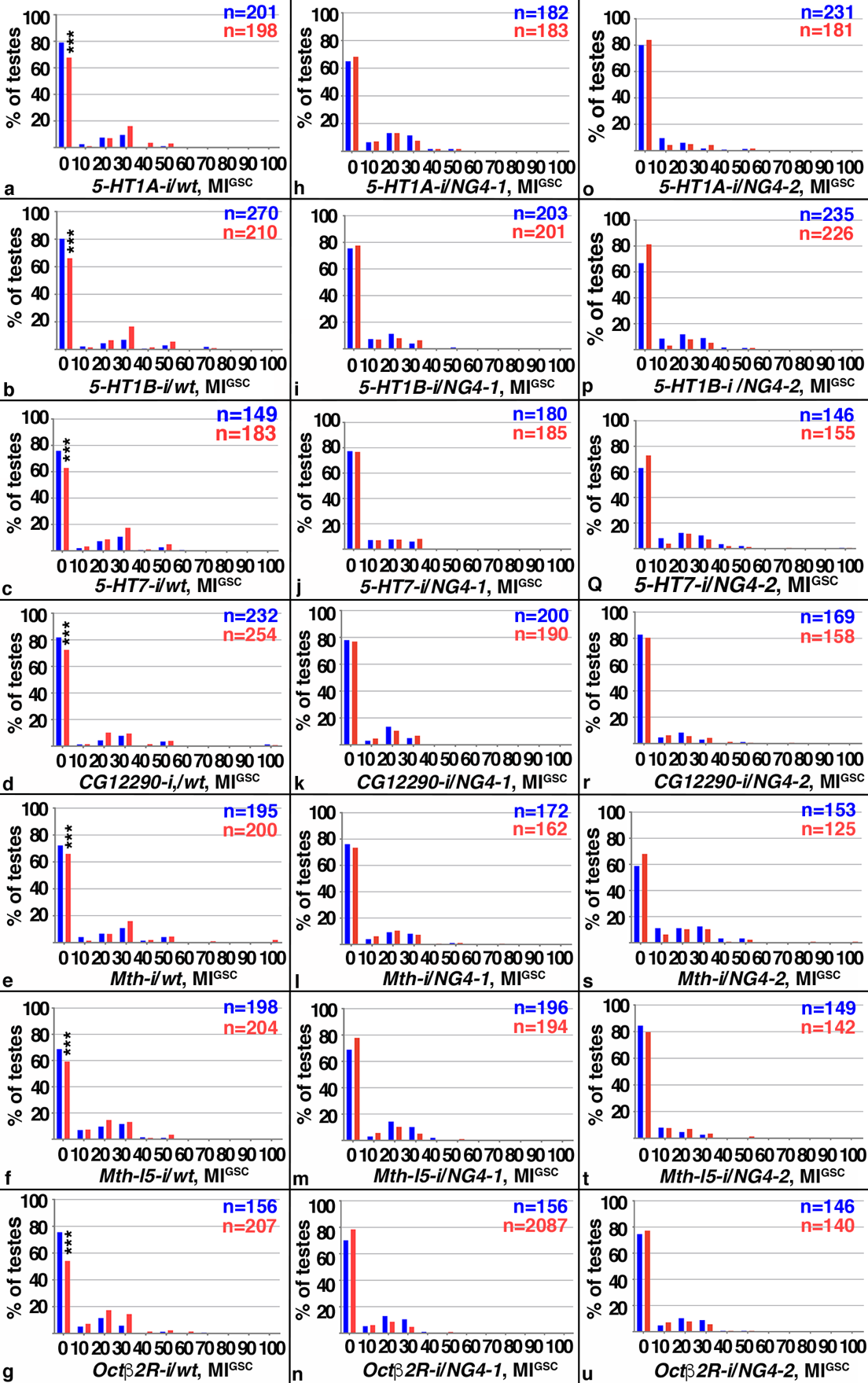
**

**Fig. S4. No change in the distribution of MI^GSC^ in response to mating was seen upon expressing RNA-*i* against seven of the GPCRs.**

a-u) FDGs showing median of bin of MI^GSC^ across populations of males on the X-axis (bin width=10) and the percentage of testes with each MI^GSC^ on the Y-axis. Blue: non-mated condition, red: mated condition, n; number of testes examined, genotypes as indicated, ***: P-value < 0.001.

a-g) Control males.

h-u) Males expressing RNA-*i* against seven GPCRs in the in the germline via (h-n) *NG4-1* or (o-u) *NG4-2*.

**Table S1. Protein- and peptide-binding receptors expressed at the testis tip**

Gene names and predicted function of receptors are listed that were found to be expressed at the testis tip, based on RNA-sequencing. Olfactory and taste receptors are excluded from this list.

| **Gene name** | **Predicted molecular function** |
| --- | --- |
| 18w | transmembrane signaling receptor activity |
| 5-HT1A | Gi/o-coupled serotonin receptor activity ; |
| 5-HT1B | Gi/o-coupled serotonin receptor activity |
| 5-HT2A | serotonin receptor activity |
| 5-HT2B | G-protein coupled amine receptor activity |
| 5-HT7 | serotonin receptor activity ; dopamine neurotransmitter receptor |
| AdoR | G-protein coupled adenosine receptor activity, Adenosine |
| AkhR | neuropeptide receptor activity, Adipokinetic hormone receptor |
| arr | low-density lipoprotein receptor activity arrow |
| b6 | neuronal pentraxin receptor activity |
| CapaR | neuropeptide receptor activity, Capability receptor |
| CCAP-R | neuropeptide receptor activity, Crustacean cardioactive peptide |
| CG1887 | scavenger receptor activity |
| CG2061 | G-protein coupled receptor activity |
| CG2736 | scavenger receptor activity |
| CG3829 | scavenger receptor activity |
| CG3921 | scavenger receptor activity |
| CG4168 | G-protein coupled receptor activity |
| CG4313 | G-protein coupled receptor activity ; |
| CG5621 | kainate selective glutamate receptor activity |
| CG5888 | transmembrane signaling receptor activity |
| CG8909 | low-density lipoprotein receptor activity |
| Cirl | latrotoxin receptor activity |
| clumsy | kainate selective glutamate receptor activity clumsy |
| crq | scavenger receptor activity, croquemort |
| cry | G-protein coupled photoreceptor activity |
| CrzR | neuropeptide receptor activity, Corazonin receptor |
| Ddr | transmembrane receptor activity |
| Dh31-R | calcitonin receptor activity |
| disp | hedgehog receptor activity |
| dome | transmembrane signaling receptor activity |
| Dop1R1 | dopamine neurotransmitter receptor activity |
| Dop1R2 | dopamine neurotransmitter receptor activity, |
| Dop2R | G-protein coupled amine receptor activity |
| DopEcR | G-protein coupled amine receptor activity |
| Dscam1 | axon guidance receptor activity |
| eater | scavenger receptor activity |
| EcR | ecdysteroid hormone receptor activity |
| emb | nuclear export signal receptor activity |
| emp | scavenger receptor activity |
| et | receptor activity |
| ETHR | G-protein coupled peptide receptor activity |
| FMRFaR | G-protein coupled receptor activity |
| fz | Wnt-activated receptor activity ; |
| fz2 | transmembrane signaling receptor activity |
| fz4 | transmembrane signaling receptor activity |
| GABA-B-R2 | G-protein coupled GABA receptor activity |
| GABA-B-R3 | G-protein coupled GABA receptor activity |
| Gfrl | glial cell-derived neurotrophic factor receptor activity |
| Gli | receptor activity |
| GluRIA | alpha-amino-3-hydroxy-5-methyl-4-isoxazole propionate selective glutamate receptor activity |
| GNBP3 | signaling pattern recognition receptor activity |
| gogo | receptor activity |
| Gpa2 | G-protein coupled receptor binding |
| Gprk1 | G-protein coupled receptor binding |
| Gprk2 | G-protein coupled receptor kinase activity |
| hec | calcitonin receptor activity |
| InR | insulin-activated receptor activity |
| lea | axon guidance receptor activity |
| Lerp | receptor activity |
| Lgr1 | G-protein coupled receptor activity |
| Lgr3 | protein-hormone receptor activity |
| Lkr | neuropeptide receptor activity |
| LpR1 | low-density lipoprotein receptor activity |
| LpR2 | low-density lipoprotein receptor activity |
| LRP1 | low-density lipoprotein receptor activity |
| mgl | low-density lipoprotein receptor activity |
| mGluR | G-protein coupled receptor activity |
| MsR1 | myosuppressin receptor activity |
| MsR2 | myosuppressin receptor activity |
| mthl1 | G-protein coupled receptor activity |
| mthl12 | G-protein coupled receptor activity, methuselah-like 12 |
| mthl13 | G-protein coupled receptor activity |
| mthl14 | G-protein coupled receptor activity |
| mthl15 | G-protein coupled receptor activity |
| mthl2 | G-protein coupled receptor activity |
| mthl5 | G-protein coupled receptor activity ; |
| mthl6 | G-protein coupled receptor activity |
| mthl7 | G-protein coupled receptor activity |
| mthl8 | G-protein coupled receptor activity |
| mthl9 | G-protein coupled receptor activity |
| mtt | G-protein coupled receptor activity, mangetout |
| N | transmembrane signaling receptor activity |
| ninaD | scavenger receptor activity |
| ninaE | G-protein coupled photoreceptor activity |
| Nmdar1 | N-methyl-D-aspartate selective glutamate receptor activity |
| Npc1a | hedgehog receptor activity |
| Npc1b | hedgehog receptor activity |
| NPFR | neuropeptide F receptor activity |
| Oamb | octopamine receptor activity |
| Oct-TyrR | G-protein coupled amine receptor activity |
| Octbeta1R | G-protein coupled amine receptor activity |
| Octbeta2R | G-protein coupled amine receptor activity |
| Octbeta3R | G-protein coupled amine receptor activity |
| Pdfr | calcitonin receptor activity |
| PK1-R | G-protein coupled receptor activity |
| PK2-R1 | G-Pprotein coupled receptor activity |
| PK2-R2 | G-protein coupled receptor activity |
| PlexA | axon guidance receptor activity |
| PlexB | semaphorin receptor activity |
| pog | glutamate receptor activity |
| Proc-R | G-protein coupled peptide receptor activity |
| ptc | hedgehog receptor activity |
| Ptr | receptor activity |
| Rdl | GABA-A receptor activity |
| Rh3 | G-protein coupled photoreceptor activity |
| Rh4 | G-protein coupled photoreceptor activity |
| Rh5 | G-protein coupled photoreceptor activity |
| Rh7 | G-protein coupled photoreceptor activity |
| rk | G-protein coupled receptor activity |
| robo3 | axon guidance receptor activity |
| Rya-R | neuropeptide receptor activity |
| Sdc | transmembrane signaling receptor activity |
| sev | transmembrane receptor protein tyrosine kinase activity, |
| SIFaR | neuropeptide receptor activity, SIFamide receptor |
| smo | G-protein coupled receptor activity |
| Snmp1 | scavenger receptor activity |
| Snmp2 | transmembrane signaling receptor activity |
| sNPF-R | neuropeptide receptor activity |
| sNPF-R | neuropeptide receptor activity |
| SPR | G-protein coupled peptide receptor activity Sex peptide |
| Sr-CI | scavenger receptor activity |
| Sr-CII | scavenger receptor activity |
| stan | G-protein coupled receptor activity |
| Tehao | transmembrane signaling receptor activity |
| TkR86C | tachykinin receptor activity |
| TkR99D | neuropeptide receptor activity |
| tkv | transforming growth factor beta receptor activity, type I |
| Tl | transmembrane signaling receptor activity |
| Toll-4 | transmembrane signaling receptor activity |
| Toll-6 | transmembrane signaling receptor activity |
| Toll-7 | transmembrane signaling receptor activity |
| Toll-9 | transmembrane signaling receptor activity |
| Tollo | transmembrane signaling receptor activity |
| Tre1 | G-protein coupled receptor activity |
| TrissinR | neuropeptide receptor activity |
| TyrR | G-protein coupled amine receptor activity |
| TyrRII | octopamine receptor activity |
| unc-5 | netrin receptor activity |
| verm | low-density lipoprotein receptor activity |
| Vmat | monoamine transmembrane transporter activity |
| wgn | tumor necrosis factor-activated receptor activity |
| yl | vitellogenin receptor activity |

**Table S2. Fertility Assay**

Male fertility was calculated based on the % of females that produced offspring after mating with males of the indicated genotype. BL#: Bloomington stock number.

| **Genotype** | **BL #** | **Male fertility** |
| --- | --- | --- |
| *OR* | N/A | 72% |
| *CS* | N/A | 62% |
| *5HT-1A-i /NG4-1* | 33885 | 75% |
| *5HT-1B-i /NG4-1* | 33418 | 61% |
| *5HT-7-i/NG4-2* | 27273 | 91% |
| *CG12290-i /NG4-1* | 31873 | 77% |
| *Mth-i /NG4-1* | 36823 | 90% |
| *Mth-l5-i /NG4-1* | 42515 | 84% |
| *Octβ2R-i /NG4-1* | 50580 | 89% |
| *5HT-1A^Δ5kb^ /5HT-1A^Δ5kb^* | 27640 | 15% |
| *5HT-1B^ΔIII-V^ /5HT-1B^ΔIII-V^* | 55846 | 30% |
